# New York Letter

**Published:** 1897-03

**Authors:** Ogden C. Ludlow

**Affiliations:** 2309 Seventh Avenue; New York


### CORRESPONDENCE.

### NEW YORK LETTER.

NEW YORK, February, 1897.

Just now, when activity in medical circles is at its height, it is somewhat difficult to choose from among the many subjects clamoring for recognition. Perhaps one that is of great interest to the general reader is that of the prevention of the spread of contagious diseases. It has long been known that our public and parochial schools were centers of much contagion, yet in the past the best efforts of the health authorities to remedy this evil have been turned to naught. A few years ago one physician in this city seriously suggested that a large number of medical inspectors be appointed, and that they should make daily visits to the schools and personally examine the children, particularly as to the condition of the throat. This proposition met with a good deal of ridicule, although the medical profession, at least, recognized its theoretical value. Recently our health department resolved to test the matter. One of their physicians had ascertained that during three weeks of last October, out of thirty-seven cases of measles investigated, thirteen could be traced to a parochial school, two to a public school, and seven to a kindergarten. This made clear the important part played by the schools in the dissemination of these diseases. It was found that one hundred and fifty school inspectors would be required, and that the funds at their disposal were so limited that the remuneration for each inspector must be limited to thirty dollars a month. This niggardly salary for physicians charged with such important duties has, as might be expected, been very severely criticised. It has been argued, and with much reason, that competent physicians would hardly be willing to undertake the task of daily inspecting the schools, if no greater inducement could be offered. Be that as it may, it has been whispered around that already there is no lack of applicants for these positions, and that the list includes at least one professor in a medical college!

Children who have been absent from the school for over two days are not to be allowed to return to their class-rooms until the medical inspector shall have satisfied himself that the child is free from all possibility of contagion. In every case of sore throat a culture is to be taken for bacteriological examination. The inspector is also required to supervise the disinfection of desks and books of children who have been sick with a contagious disease. While there is much reason to believe that it will be found exceedingly difficult to carry out these contemplated measures in actual practice, by all means let the good work go on. Many a grief-stricken father and mother has cause to curse the day that they ever allowed their dear little ones to attend these schools.

But let us consider this subject of the prevention of the spread of contagious diseases from another standpoint—that of the home. And first of all, are we, physicians, entirely blameless? Have we never been guilty of carrying contagion to our own, or our neighbor's fireside? Doubtless many will answer indignantly "no"; yet surely there are well authenticated cases of this kind on record. Perhaps if we are not ourselves guilty, we can recall some medical friend who has had reason to remember such a sin of omission; at any rate, a little introspection of this kind will do no harm. Having satisfied ourselves on this point, we should consider carefully the measures to be taken at the patient's house to prevent the dissemination of the disease. These have been very plainly set forth in a recent paper read before the New York Academy of Medicine by Dr. Henry Dwight Chapin. After emphasizing the necessity for free ventilation in the sick-room, and the removal of superfluous furniture and hangings, he calls attention to the fact that even among the poorest persons the discharges from the patient can be easily disposed of by directing that they shall be received on pieces of cheese-cloth, and that these shall be immediately burned. This is a little practical point that by reason of its littleness is often entirely overlooked, yet it is a matter of vital importance to the attendant on a case of diphtheria. Before bacteriology came to the aid of the clinician, he was wont to wait a few days after the disappearance of the diphtheritic membrane, and then pronounce it safe for the patient to mingle with healthy persons. But this is all changed now, and, in this city at least, it may be necessary to wait for a number of weeks after convalescence has been apparently established before the health authorities will give a clean bill of health. Naturally, this often entails great hardship, and though the general wisdom of the rule is not denied, a little discretionary power would afford much relief. And just here it is well to note what Dr. Chapin says about the cases in which the diphtheria bacilli persist for a long time: "I have, however, never seen infection disseminated in this way after a week or two, particularly if disinfectant solutions have been used systematically, in the form of gargles or irrigations." He adds, that an excellent and cheap disinfectant solution for general use in the sick-room may be made by dissolving four ounces of sulphate of zinc and two ounces of common salt in a gallon of water. After the person has recovered, the room should be disinfected by sulphur fumigation or the use of strong solutions of bichloride of mercury, and the bedding or other heavy fabrics should be exposed for many hours to the sun and air.

Another subject that has received a large share of attention here this winter is the serum diagnosis of typhoid fever by Widal's blood test. Our health department has given a praiseworthy impetus to this study by offering to examine, free of charge, all specimens of blood sent to them by physicians. Dr. W. H. Park, who has been indefatigable in these studies, presented some of his results the other evening at a medical meeting. He stated that while the method of employing dried blood was convenient, as only two or three drops of blood were required, and the specimen could be easily transported to the laboratory, the test was not always as satisfactory as where serum was used. According to his experience, if dried blood be used, the reaction must be immediate, and must be completed inside of fifteen minutes if one would draw reliable conclusions from the test. When the reaction from the dried blood fails or is equivocal, it is better to repeat the test, using serum obtained from a blister. The reaction was obtained the first week of typhoid fever in 70 per cent. of the cases, in the second week in 69 per cent., in the third week in 75 per cent., and in the fourth week in 92 per cent. of the cases examined. It is very important, in applying this test to note the dilution at which the reaction is obtained. Thus, in the investigation referred to, in not a single instance did the blood from a healthy person give the typhoid reaction in *one-tenth dilution.* The blood from two persons suffering from other diseases than typhoid fever gave the typhoid reaction, but in these the reaction did not appear with a higher dilution than one-tenth, which is not the case in typhoid fever. Dr. J. W. Brannan states that he believed that in 95 per cent. of cases of typhoid fever the reaction could be obtained about the eighth day, but that it was not a test that could be successfully applied by the average general practitioner. Considerable doubt has been expressed regarding the trustworthiness of the test in many instances, owing to the possibility of the reaction being due to a previous attack of typhoid fever. Experience seems to show that it is quite rare for the reaction to be observed a year after an attack of typhoid fever, and Dr. Brannan says that in the cases that he has observed the reaction has not been present later than four or five months after convalescence from typhoid fever. Several of those who have taken part in the recent discussion of this subject have stated that in rare instances the reaction will be alternately present and absent for a number of days. Dr. Elsberg, who has been carefully observing an epidemic of typhoid fever at the Mount Sinai Hospital, states that although in a large majority of the cases he has obtained the reaction between the eighth and twelfth days, two cases did not give the reaction for typhoid fever until the twenty-sixth and twenty-eighth days respectively.

It goes without saying that our surgeons have not felt that they were "up to date" this season unless they could occasionally put in evidence X ray photographs. Although I have had an opportunity to see much excellent work of this kind during the past year, I have only seen one patient who at the time presented the evidence of the injurious effects of these rays. This was a patient presented by Dr. Willy Meyer. In order to secure X ray pictures through this man's skull, and so locate two bullets that he had discharged into his brain, it became necessary to give two or three rather long exposures. A few days after the last seance the hair fell out of the side of the head that had been directly exposed to source of the X rays, and when I saw him the scalp over that side of the head was completely denuded of hair. When presenting this case, Dr. Meyer made the remark that, so far as we knew, such an unpleasant result was only temporary. This statement, however, is entirely opposed to the experience of some observers. I also know of an electrician who suffered from a very persistent dermititis as a result of prolonged exposure to X rays. In this case it is worthy of note that eight or ten days elapsed before the development of the dermatitis. Regarding these occasional untoward effects of the X rays, it may be said, I think, that they have, so far, only been observed after a very long exposure, and when the vacuum tube has been placed very close to the subject. While, therefore, such results are possible, and may occur under extraordinary circustances, they do not interfere in any way with the legitimate use of this valuable auxiliary in surgery.

While speaking of the aid rendered by these skiagraphs, I am reminded of a case of bone-grafting exhibited by Dr. A. M. Phelps. The lady had been treated long and unsuccessfully by the usual methods for a fracture of both bones of the forearm. Dr. Phelps finally essayed to make the fracture unite by means of bone-grafting, and having succeeded in this endeavor, he had an X-ray picture taken of the forearm, and brought it along with the patient as proof that he had produced the cure by the method claimed. This consisted in removing a portion of the fore-leg of a young pup, cutting the bone up into fine spiculæ, and inserting these close together underneath the periosteum at the seat of the fracture. The periosteum was then stitched over them, and the layers of bone-grafts formed a sort of coaptation splint about the shaft of the bone. He said that by using these chips of *vascular* bone, instead of decalcified bone-chips, as Senn does, he is able to start up centers of ossification. During the past five years he has treated all cases in this way. In the early ones the technique was very imperfect and they failed, but perfect union was obtained in the remaining eight cases.

An ingenious microtome has been invented and put upon the market, by Dr. Sidney Yankauer, of this city. It is constructed on entirely new principles, and, in addition to its principal merit—that of cheapness—it is efficient, the work done by it comparing favorably with that of microtomes costing fifty dollars or more. In the new instrument the specimen remains stationary, and the plane of the knife is continually lowered. It will cut sections in celloidin having a thickness of fifteen microns, and in paraffin sections having a thickness of only three microns. It is not, however, well adapted to making serial sections. The inventor demonstrated its working the other evening before the Pathological Society. The instrument can be procured from Tiemann & Co. for ten dollars.

OGDEN C. LUDLOW, M.D.

*2309 Seventh Avenue.*

---

### SURGERY OF EMPYEMA.

Rafferty (*Medical Record*, September 26, 1896,) says: "All operative measures in the treatment of empyema have for their chief end two objects: first, to evacuate the pus or other fluid contained in the cavity; and, second, the obliteration of the cavity by bringing together its walls. The latter is best done by that method which closes the cavity by expansion of the lung, and not by retraction of the bony thorax."

With these ends in view he recommends, first, a resort to simple aspiration, conducted with great attention to the carrying out of all antiseptic details; if the fluid is serous, this is probably all that will be required. However, if the fluid is purulent, a reaccumulation is likely to occur, in which case either simple incision or puncture and permanent drainage should be practiced. If neither of these methods gives sufficient drainage, a small portion of rib may be excised.

If the lung is bound down by adhesions so that expansion is impossible, then Estlander's operation is clearly indicated, in which case there must be sufficient excision of ribs so that by collapse of the bony thorax the costal pleura will be brought in contact with the pleura covering the lung.—*Codex Medicus.*

